# Prevalence of Osteoarthritis Using Cone Beam in Patients at the University of Cuenca

**DOI:** 10.1002/cre2.70344

**Published:** 2026-04-26

**Authors:** Andrea Michelle Astudillo Uchupaille, Iván Andrés Palacios Astudillo, Yulissa Raquel Abad Salinas, Valeria Paulina Romero Rodríguez, Wilson Daniel Bravo Torres

**Affiliations:** ^1^ Department of Oral Rehabilitation and Implant‐Supported Prosthetics University of Cuenca Cuenca Ecuador; ^2^ Department of Imaging University of Cuenca Cuenca Ecuador

**Keywords:** cone beam computed tomography, osteoarthritis, temporomandibular joint, temporomandibular joint disorders

## Abstract

**Objective:**

To determine the prevalence of temporomandibular joint (TMJ) osteoarthritis using Cone Beam Computed Tomography (CBCT) in patients from the Faculty of Dentistry at the University of Cuenca during the period 2017–2023.

**Methods:**

A descriptive, cross‐sectional, quantitative study was conducted, evaluating 201 computed tomography scans obtained from the Imaging Department of the Faculty of Dentistry at the University of Cuenca from 2017 to 2023. A total of 402 temporomandibular joints were analyzed for imaging characteristics such as subarticular bone erosion, articular flattening, osteophytes, subchondral cysts, and sclerosis.

**Results:**

The prevalence of OA was 31.1%, with 8.5% of male patients and 22.6% of female patients diagnosed. Among the joints analyzed, 27.3% showed osteophytes, 7.4% showed erosion, and 17.4% showed pseudocysts.

**Conclusion:**

Less than half of the joints analyzed showed signs of OA, with females being the most affected and ages 18 to 28 years being the most common.

## 1 Introduction

Temporomandibular disorders (TMDs) are a complex group of pathologies involving masticatory muscles, the temporomandibular joint (TMJ), and associated structures. These disorders have a multifactorial etiology and occur due to mechanical and biological events, potentially affecting the morphology of the TMJ's hard tissue components (Walewski et al. 2019). Among these, osteoarthritis (OA) is one of the primary TMDs (Kothari et al. 2016; Mélou et al. 2022).

OA is characterized by the chronic degeneration of bone, cartilage, and soft tissues within and around the joint, leading to joint pain due to altered peripheral and central pain processing mechanisms. OA can affect the TMJ by altering the morphological characteristics of cartilage, subchondral bone, and synovial membrane, resulting in TMJ remodeling and cartilage abrasion (Bansal 2016; Derwich et al. 2020a).

According to the World Health Organization (WHO), the global prevalence of OA ranges from 28% to 38%, with incidence increasing with age. OA is primarily caused by the degeneration of collagen and proteoglycans in cartilage, leading to fibrillation, erosion, and cracking of the cartilage's surface layer (Pantoja et al. 2019). This process extends to deeper layers, eventually forming erosions. The TMJ's articular surface exhibits notable adaptability. While hyaline cartilage in weight‐bearing joints is more resistant to compressive load, TMJ fibrocartilage is better suited to resist shear forces. Disease occurs when functional demands exceed TMJ adaptability or when an individual is predisposed to maladaptive responses (Valesan et al. 2021; Massilla Mani and Sivasubramanian 2016).

The cardinal features of TMJ OA are both clinical and radiographic. Clinical characteristics include tenderness in the joint region, pain during movement such as mouth opening or lateral excursion, and crepitus. Diagnostic imaging via CT offers superior visualization of bone changes, providing multidimensional views with greater reliability and accuracy compared to panoramic radiographs (Massilla Mani and Sivasubramanian 2016; Koç 2020). Common imaging features include cortical bone erosion, flattening of articular compartments, and productive bone changes like sclerosis and osteophytes. These signs represent various stages of disease progression, with erosive lesions and joint space narrowing indicating early changes, while sclerosis, flattening, subchondral cysts, and osteophytes indicate late‐stage changes (Verghese et al. 2023; Bäck et al. 2017).

This study aims to determine the prevalence of TMJ OA using CBCT in patients from the Faculty of Dentistry at the University of Cuenca from 2017 to 2023.

## 2 Materials and Methods

### 2.1 Study Population

This quantitative, descriptive, and cross‐sectional study analyzed CBCT scans from the Imaging Department of the Faculty of Dentistry at the University of Cuenca from 2017 to 2023.

A simple random sampling technique was used. From a total population of 1074 CBCT scans, a sample of 201 scans was determined using a finite population formula with a 95% confidence level and a 5% margin of error. A total of 402 temporomandibular joints were analyzed.

**Inclusion Criteria**: CBCT scans in occlusion, including both maxillae and TMJs.

**Exclusion Criteria**: Scans with artifacts impeding TMJ observation, movement artifacts, or patients undergoing orthodontic treatment with fixed appliances.

### 2.2 Data Collection

Data were collected through CBCT evaluations. A data collection sheet was used, comprising patient demographics and imaging findings for the right and left TMJs.

CBCT scans were performed using an Accuitomo 170 3D machine (J. Morita) following a standardized scanning protocol (kVp, mA, FOV 17 × 21 cm, voxel size 330 microns, approximate effective dose = mSv). Multiplanar reconstructions and analyses were conducted using i‐Dixel V 2.2.0.3 software.

A licensed imaging specialist performed all scans, and calibration was conducted by field experts. The principal investigator's inter‐observer agreement was evaluated using the Kappa coefficient.

Right and left TMJs were evaluated using lateral reconstructed images in sections perpendicular to the condyle's long axis, coronal sections parallel to the condyle's long axis, and axial images of the joint. Reconstruction curves were traced using the Curve MPR module to obtain parasagittal and paracoronal sections for both sides. Joints were analyzed by comparing each side.

OA diagnosis was based on Wilkes' classification (Table 1), with measurements obtained using the “measurements and overlays tool.

**Table 1:** Wilkes classification for OA diagnosis.

**Grade 1**	Osteophyte: The largest length of the osteophyte is < 2 mm when measured from the tip of the osteophyte to the expected contour of the condyle as seen in the corrected sagittal section. Erosion: The largest dimension of the erosion is <2 mm in depth and width, and the erosion is limited to a single occurrence. Subcortical pseudocyst: The largest dimension of the pseudocyst is < 2 mm in depth and width, and the pseudocyst is limited to a single occurrence.
**Grade 2**	Osteophyte: The largest length of the osteophyte is > 2 mm when measured from the tip of the osteophyte to the expected contour of the condyle as seen in the corrected sagittal section. Erosion: The largest dimension of the erosion is > 2 mm in depth and width, or there is more than one erosion of any size. Subcortical pseudocyst: The largest dimension of the pseudocyst is ≥ 2 mm, or there is more than one pseudocyst of any size. Two or more imaging signs of grade 1 degenerative joint disease.

If no findings consistent with OA are found, the patient is considered to be without OA. To diagnose the aforementioned characteristics, the following incidental findings must be taken into account:

- **Subcortical cyst**: A cavity beneath the articular surface that deviates from normal medullary patterns.
- **Erosion**: A loss of continuity in the articular cortex.
- **Osteophyte**: Marginal hypertrophy with sclerotic edges and angular exophytic formation.

### 2.3 Statistical Analysis

Data were processed using SPSS version 25. Kolmogorov‐Smirnov tests assessed data normality, Chi‐square tests associated primary variables with covariates, and Pearson's correlation coefficient assessed significant relationships between primary study variables and sex and age (*p* < 0.05).

### 2.4 Ethical Considerations

This study presented no ethical conflicts, as it involved only imaging analysis without direct patient contact. Confidentiality and anonymity were maintained. Ethical approval was obtained from the Human Research Ethics Committee (CEISH) at the University of Cuenca.

## 3 Results

Of the 402 temporomandibular joints analyzed, 27.3% had osteophytes, 7.4% had erosion, and 17.4% had pseudocysts. According to the participants' sex, it was observed that 7.4% of men and 19.9% of women had osteophytes. Additionally, 2.0% of men and 5.5% of women had erosion. Furthermore, 4.7% of men and 12.7% of women had pseudocysts. An association was observed between sex and the presence of osteophytes, and between sex and OA, with *p*‐values of 0.051 and 0.031, respectively (Table 2).

**Table 2:** Incidental findings according to sex.

Incidental findings	Male				Female				Total				Chi square	*p‐*value
	Yes		No		Yes		No		Yes		No			
	*n*	%	*n*	%	*n*	%	*n*	%	*n*	%	*n*	%		
Osteophyte	30	7.4	110	27.3	80	19.9	182	45.3	110	27.3	292	72.6	3.806	0.051
Erosion	8	2.0	132	32.8	22	5.5	240	59.7	30	7.4	372	92.6	0.951	0.329
Pseudocyst	19	4.7	121	30.1	51	12.7	211	52.5	70	17.4	332	82.6	2.204	0.138
Osteoarthritis	34	8.5	106	26.3	91	22.6	171	42.5	125	31.1	277	68.9	4.648	0.031

According to the prevalence of OA, it was found that 31.1% of the analyzed CT scans were diagnosed with OA. It was observed that of the total patients with OA, 8.5% were men and 22.6% were women. Additionally, 26.9% were aged between 18 and 28 years, 2.5% between 29 and 38 years, 0.7% between 39 and 48 years, and 1.0% between 59 and 68 years. Furthermore, of the patients with OA, 15.2% had it in the right TMJ, and 15.9% in the left TMJ. A statistically significant association was found between the presence of OA and the variables sex and age, with a *p*‐value of 0.031 and 0.004, respectively (Table 3).

**Table 3:** Osteoarthritis and its relationship with sex, age, and laterality.

Characteristics	Yes		No		Total		Chi square	*p‐*value
	*n*	%	*n*	%	*n*	%		
**Sex**								
Female	91	22.6	171	42.5	262	65.2	4.648	0.031
Male	34	8.5	106	26.3	140	34.8		
**Age**								
18 to 28	108	26.9	220	54.7	328	81.6	15.377	0.004
29 to 38	10	2.5	44	10.9	54	13.4		
39 to 48	3	0.7	9	2.2	12	3.0		
49 to 58	0	0.0	4	1.0	4	1.0		
59 to 68	4	1.0	0	0.0	4	1.0		
**Laterality**								
Right	61	15.2	140	3.5	201	50.0	0.104	0.747
Left	64	15.9	137	34.1	201	50.0		

Of the total cases of OA, 95.3% were bilateral, meaning they affected both the right and left temporomandibular joints, 4.7% affected only the left joint, and 0% affected the right joint (Table 4).

**Table 4:** Prevalence of osteoarthritis and its relationship with laterality.

Laterality	Osteoarthritis	
	*n*	%
Right	0	0.0
Left	3	4.7
Bilateral	61	95.3

Regarding laterality, it was found that, of the total patients with osteophytes, 13.7% had them in the right TMJ and 13.7% in the left TMJ. Of the patients with erosion 3.5% had it in the right side and 4.0% in the left side. In contrast, of the patients with pseudocysts, 8.9% had them in the right TMJ and 8.5% in the left side. No association was observed between laterality and incidental findings (Table 5).

**Table 5:** Incidental findings according to laterality.

Incidental findings	Right				Left				Total				Chi square	*p*–value
	Yes		No		Yes		No		Yes		No			
	*n*	%	*n*	%	*n*	%	*n*	%	*n*	%	*n*	%		
Osteophyte	55	13.7	146	36.3	55	13.7	146	36.3	110	27.3	292	72.6	0.000	1.000
Erosion	14	3.5	187	46.5	16	4.0	185	46.0	30	7.4	372	92.6	0.144	0.704
Pseudocyst	36	8.9	165	41.0	34	8.5	167	41.5	70	17.4	332	82.6	0.069	0.793
Osteoarthritis	61	15.2	140	3.5	64	15.9	137	34.1	125	31.1	277	68.9	0.104	0.747

Regarding age, of the patients who had osteophytes, 25.6% were aged between 18 and 28 years, 2.0% were aged between 29 and 38 years, 0.7% were aged between 39 and 48 years, and 1.0% were aged between 59 and 68 years. In contrast, of the patients with erosion, 5.9% were aged between 18 and 28 years, 1.0% were aged between 29 and 38 years, 0.2% were aged between 39 and 48 years, and 0.2% were aged between 59 and 68 years. Similarly, of the patients who had pseudocysts, 15.2% were aged between 18 and 28 years, 2.2% were aged between 29 and 38 years, 0.2% were aged between 39 and 48 years, and 0.7% were aged between 59 and 68 years. A statistical association was found between age and the presence of osteophytes, age and pseudocysts, and age and OA, with *p*‐values of 0.002, 0.009, and 0.004, respectively (Table 6).

**Table 6:** Incidental findings according to age.

Incidental findings	18 to 28				29 to 38				39 to 48				49 to 58					59 to 68					Total				Chi square	*p‐*value
	Yes		No		Yes		No		Yes		No		Yes		No			Yes		No			Yes		No			
	*n*	%	*n*	%	*n*	%	*n*	%	*n*	%	*n*	%	*n*	%	*n*	%	*n*		%	*n*	%	*n*		%	*n*	%		
Osteophyte	95	25.6	233	58.0	8	2.0	46	11.4	3	0.7	9	2.2	0	0.0	4	1.0	4		1.0	0	0.0	110		27.3	292	72.6	16.859	0.002
Erosion	24	5.9	304	75.6	4	1.0	50	12.4	1	0.2	11	2.7	0	0.0	4	1.0	1		0.2	3	0.7	30		7.4	372	92.6	2.128	0.712
Pseudocyst	61	15.2	267	66.4	5	2.2	49	12.2	1	0.2	11	2.7	0	0.0	4	1.0	3		0.7	1	0.2	70		17.4	332	82.6	13.572	0.009
Osteoarthritis	108	26.9	220	54.7	10	2.5	44	10.9	3	0.7	9	2.2	0	0.0	4	1.0	4		1.0	0	0.0	125		31.1	277	68.9	15.377	0.004

Regarding the Wilkes Classification, it was found that 5.2% of female patients and 2.2% of male patients had grade 1, and 17.4% of female patients and 6.2% of male patients had grade 2. Regarding age, 6.4% of patients aged 18 to 28 years and 1.0% of patients aged 39 to 48 years had grade 1, and 20.4% of patients aged 18 to 28 years, 1.4% aged 29 to 38 years, 0.7% aged 39 to 48 years, and 1.0% aged 59 to 68 years had grade 2. Concerning laterality, 3.2% of patients had grade 1 in the right TMJ, and 4.2% had grade 1 in the left TMJ; additionally, 11.9% had grade 2 in the right TMJ and 11.7% had grade 2 in the left TMJ. An association between age and the Wilkes Classification was observed (Table 7).

**Table 7:** Wilkes classification.

Characteristics	Without OA		Grade 1		Grade 2		Total		Chi square	*p*‐value
	*n*	%	*n*	%	*n*	%	*n*	%		
**Sex**										
Female	171	42.53	21	5.2	70	17.4	262	65.2	4.784	0.091
Male	106	26.4	9	2.2	25	6.2	140	34.8		
**Age**									21.037	0.007
18 to 28	220	54.7	26	6.4	82	20.4	328	81.6		
29 to 38	44	10.9	4	1.0	6	1.4	54	13.4		
39 to 48	9	2.2	0	0.0	3	0.7	12	3.0		
49 to 58	4	1.0	0	0.0	0	0.0	4	1.0		
59 to 68	0	0.0	0	0.0	4	1.0	4	1.0		
**Laterality**										
Right	140	34.8	13	3.2	48	11.9	201	50.0	0.576	0.75
Left	137	34.1	17	4.2	47	11.7	201	50.0		

Of the total computed tomography scans analyzed, 65.2% corresponded to female patients and 34.8% to male patients. Among these, 81.6% of the patients were aged between 18 and 28 years, 13.4% were aged between 29 and 38 years, 3.0% were aged between 39 and 48 years, 1.0% were aged between 49 and 58 years, and 1.0% were aged between 59 and 68 years (Table 8).

**Table 8:** Characterization of the study population.

	*n*	%
**Sex**		
Female	262	65.2
Male	140	34.8
**Age**		
*Age range*		
From 18 to 28	328	81.6
From 29 to 38	54	13.4
From 39 to 48	12	3.0
From 49 to 58	4	1.0
From 59 to 68	4	1.0

A very low positive correlation was demonstrated between the prevalence of OA and the sex variable, with a Pearson's *r* of 0.108 and a significant correlation between these two variables was found with a *p*‐value of 0.031. However, statistical significance was found between the prevalence of OA and the age variable, with a *p*‐value of 0.495 (Table 9).

**Table 9:** Pearson correlation of osteoarthritis with sex and age.

Osteoarthritis	Sex	Age
Pearson correlation (*r*)	0.108	0.034
Bilateral significance (*p*)	0.031	0.495

## 4 Discussion

OA is the most common disease affecting temporomandibular joints, typically a slow‐progressing disease that affects the entire joint, including the articular cartilage, subchondral bone, ligaments, synovial membrane, and even the surrounding muscles. The etiology of degenerative changes in the joints is complex. It has been suggested that degenerative changes occur as a result of altered remodeling of the temporomandibular joint (Derwich et al. 2020a; Park et al. 2023).

In this study, a total of 402 temporomandibular joints were analyzed, of which 27.3% showed osteophytes, 17.4% showed pseudocysts, and 7.4% showed erosion. These results differ from those published by Tsai et al. (2020), where the authors found that superficial erosion was the incidental finding with the highest incidence (95%), followed by osteophytes, which had the second highest incidence (80%). They also identified the presence of sclerosis and subcortical cysts in 50% and 25% of the joints, respectively.

Similarly, Arayasantiparb et al. (2020) conducted a study where they found that CBCT radiographic imaging showed flattening in 127 joints (89.4%), sclerosis in 22 joints (15.5%), subchondral cyst formation in 13 joints (9.2%), erosion in 97 joints (68.3%), and osteophytes in 124 joints (87.3%). Furthermore, the authors demonstrated a statistically significant correlation between crepitus and four radiological features, including sclerosis, subchondral cyst formation, erosion, and osteophyte formation.

Regarding sex, in this study, it was observed that females were more affected than males, with 19.9% of women presenting osteophytes, 5.5% erosion, 12.7% pseudocysts, and 22.6% OA. A statistically significant association was also found between sex and the presence of osteophytes, and between sex and OA with *p*‐values of 0.051 and 0.031, respectively. These results are consistent with those obtained by Song et al. 2020), where the authors conducted a study evaluating temporomandibular joint changes using CT scans, analyzing a total of 152 joints. They found that women had a higher prevalence of degenerative changes and OA compared to men. Additionally, they observed that most patients with OA were in their second decade of life.

Singh et al. (2021) conducted a study evaluating incidental findings in temporomandibular joints using cone beam CT scans. They found that condylar flattening and hyperplasia were significantly greater in males than in females (*p*: 0.001). The authors also found that osteophytes were more common in women (3.3% vs. 2.4%) than in men, which was significant with a *p*‐value of < 0.001. They further noted that there were no gender differences in terms of hypoplasia, soft tissue calcifications, cysts, or sclerosis. OA was not found in any of the scans.

In addition, Derwich et al. (2020b), in their research analyzed 210 temporomandibular joints from 105 patients. They found a significant correlation between the right and left temporomandibular joints concerning the number of OA changes (*p* = 0.0001). Among all the joints analyzed, articular surface flattening was the most common type of OA change, identified in 90% of cases. Regarding other OA changes, superficial erosion was present in 41.9% of cases, subcortical sclerosis in 33.3%, osteophytes in 27.1%, subcortical cysts in 11.0%, and generalized sclerosis in 1% of all joints.

Similarly, Alzahrani et al. (2020) conducted a study where they evaluated incidental findings of OA in the temporomandibular joint. They found a significant correlation between sex and the presence of OA (*p* < 0.05), with female patients having a higher prevalence of OA than male patients. Most patients with TMJ‐OA were between 50 and 59 or 60 and 69 years old. Regarding the relationship between TMJ‐OA and age, the prevalence of TMJ‐OA began to increase in the 40 to 49 age group. Specifically, 94.4% of patients aged 40 to 49 years, 97.7% of patients aged 50 to 59 years, 97.5% of patients aged 60 to 69 years, 95.8% of patients aged 70 to 79 years, and 100% of patients aged 80 to 89 years presented structural changes indicative of TMJ‐OA.

Regarding the Wilkes Classification, in this study it was found that 7.4% of OA patients presented grade 1 and 23.6% presented grade 2. These results differ from those published by Ottersen et al. (2019), where the authors found that 68% of OA joints were classified as grade 2 and 32% as grade 1. They also found osteophytes ≥ 2 mm in 61% of grade 2 joints.

Furthermore, this study showed a very low correlation between OA and sex, and OA and age. In contrast, Singh et al. (2021) calculated correlations between incidental findings and gender and age group, demonstrating that incidental findings were significantly correlated with gender (*r*: 0.553) and age (*r*: 0.314), with statistically higher correlations compared to this study.

## 5 Conclusion

After analyzing a total of 402 temporomandibular joints using computed tomography scans, it was found that osteophytes were the most common incidental finding in these patients, followed by pseudocysts and erosion. A statistical association was observed between age and the presence of osteophytes and pseudocysts. Additionally, according to the prevalence of osteoarthritis, it was obtained that 31.1% of the CT scans analyzed were diagnosed with osteoarthritis. Observing that of the total number of patients with osteoarthritis, 8.5% were men and 22.6% were women.

## 6 Biases and Limitations

Key limitations of the CBCT‐based TMJ osteoarthritis prevalence study at the University of Cuenca include:

- **Sample Size and Selection Bias**: A sample of 201 CBCT scans from 1074 patients may not represent the general population.
- **Exclusion Criteria**: Scans with artifacts or orthodontic treatment history were excluded, potentially limiting generalizability.
- **Age Distribution**: 81.6% of participants were aged 18–28, underrepresenting older populations where OA prevalence increases.
- **Gender Imbalance**: Women comprised 65.2% of the sample, potentially skewing gender‐specific conclusions.
- **Diagnostic Criteria**: Inter‐radiologist variability in interpreting Wilkes classification features may affect consistency.
- **Cross‐Sectional Design**: Limits causal or longitudinal analysis of OA progression.
- **Uncontrolled Confounders**: Lifestyle, genetics, and comorbidities were not analyzed.

## Author Contributions

**Andrea Michelle Astudillo Uchupaille:** conceptualization, methodology, investigation, formal analysis, visualization, writing – original draft. **Iván Andrés Palacios Astudillo:** conceptualization, methodology, project administration, writing – review and editing, supervision. **Yulissa Raquel Abad Salinas:** conceptualization, methodology, supervision, writing – review and editing. **Valeria Paulina Romero Rodríguez:** conceptualization, methodology, supervision, writing – review and editing. **Wilson Daniel Bravo Torres:** conceptualization, methodology, supervision, writing – review and editing.

## Funding

The authors have nothing to report.

## Conflicts of Interest

The authors declare no conflicts of interest.

## Data Availability

The data that support the findings of this study are available from the corresponding author upon reasonable request.
